# Cytokine array analyses and osteogenic potential of plasma from patients with traumatic brain-/spinal cord injury: predictive markers of heterotopic ossification?

**DOI:** 10.1007/s00068-026-03123-6

**Published:** 2026-02-23

**Authors:** Ole Somberg, Jan Gessmann, Elvira Peter, Marina Breisch, Matthias Königshausen, Thomas Schildhauer, Manfred Köller, Thomas Rosteius

**Affiliations:** 1 Department of General and Trauma Surgery, BG University Hospital Bergmannsheil, Buerkle-de-la-Camp- Platz 1, 44789 Bochum, Germany; 2 Department of Surgical Research, BG University Hospital Bergmannsheil, Buerkle-de- la- Camp-Platz 1, 44789 Bochum, Germany

**Keywords:** Spinal cord injury, Traumatic brain injury, Heterotopic ossification

## Abstract

**Aims:**

Traumatic brain injuries (TBI) and spinal cord injuries (SCI) are associated with an increased risk to develop heterotopic ossifications (HO). Aim of the study was to analyze biochemical markers and osteogenic differentiation capacity of patients’ plasma during the early posttraumatic period to assess the risk for HO.

**Methods:**

To evaluate predictive markers of HO in response to TBI/SCI, comparative cytokine array analyses of plasma obtained from patients with TBI/SCI were performed to identify expressed proteins. The expression of 105 proteins in plasma of patients with TBI (*n* = 9) and SCI (*n* = 9) was compared to patients with multiple trauma (MT; *n* = 9) and controls (norm). Additionally, the effect of patients’ plasma on osteogenic differentiation of bone marrow-derived human mesenchymal stem cells (MSC) was tested by in vitro cell assay.

**Results:**

Cytokine array analyses showed that plasma of TBI-group had a different protein expression profile compared to SCI-, MT- or norm-group. TBI-group exhibited the greatest number of up-regulated proteins and the greatest magnitude of alterations. Conversely, MT-group showed the greatest number of down-regulated proteins. In vitro cell assay showed that MSC exhibited a stronger capacity for osteogenic differentiation after treatment with plasma of TBI-/SCI-group with clinical signs of HO, than TBI-/SCI-group without HO or MT- and norm-group.

**Conclusion:**

This pilot study demonstrates distinct plasma factors in SCI/TBI patients and suggests a possible correlation between HO development and enhanced in-vitro osteogenesis of MSCs. The applied osteogenic differentiation assay could therefore represent a simple and reproducible tool to predict early stages of HO and to identify patients at risk. However, these findings do not allow causal conclusions, and further studies are required to clarify the underlying mechanisms and the predictive value of the assay.

**Level of evidence:**

III.

## Introduction

Heterotopic ossification (HO) refers to the abnormal formation of bone within the soft tissues outside the skeletal system. HO is commonly classified according to Brooker, who described its increased incidence following total hip arthroplasty. The classification distinguishes four grades, ranging from isolated bone islands to complete ankylosis of the joint [1]. In addition to its increased incidence following certain procedures, HO may also develop in association with genetic disorders such as fibrodysplasia ossificans progressiva (FOP), musculoskeletal trauma, or as a consequence of neurogenic causes including traumatic brain injury (TBI), spinal cord injury (SCI), stroke, or cerebral anoxia. HO related to neurological injury is commonly referred to as neurogenic heterotopic ossification (NHO) [2, 3]. NHO was first described after World War I, when it was observed in soldiers who had sustained traumatic SCI or TBI. These early clinical observations highlighted a clear association between severe neurologic and musculoskeletal trauma and the ectopic bone formation within soft tissues [4]. HO develops in approximately 4% to 23% of patients following TBI [5–7]. In individuals with SCI, the prevalence is substantially higher, ranging between 40% and 53% [5, 8–10]. Clinical symptoms of HO typically emerge within 3 to 4 weeks post-injury and most commonly affect the shoulder, hip, or knee joints. This pathological bone formation is associated with severe pain, swelling, joint deformity and rapid decline in joint mobility, often leading to substantial functional impairment and reduced quality of life [11]. Despite its clinical relevance, the molecular and cellular mechanisms underlying HO after TBI or SCI remain incompletely understood [9].

This study therefore aimed to identify potential plasma derived biomarkers and risk factors associated with the development of symptomatic HO in patients with TBI or SCI, using cytokine array analyses, in order to detect patients at risk at an early stage and enable timely intervention. By preventing HO, and therefore pain and immobility, this could help preserve quality of life in affected individuals.

## Methods

This study was approved by the local Ethics Committee of the Faculty of Medicine at Ruhr University Bochum (18-6509-BR). A total of nine patients with TBI, nine with SCI, nine with multiple trauma (MT), and a control group of four healthy volunteers were included. HO was defined as radiographic signs of ectopic bone formation within soft tissue or muscle in combination with clinical signs of HO observed within six months following trauma.

### Demographic and clinical characteristics of patients with SCI, TBI, and MT

Collected data on demographic and clinical characteristics included age, sex, Glasgow Coma Scale (GCS), Injury Severity Score (ISS), type and mechanism of injury, comorbidities, and duration of hospitalization. In all patients HO screening was performed using ultrasound and additional Computed tomography (CT) or Magnetic resonance imaging (MRI) imaging.

### Collection of human plasma

Whole blood samples were collected within 72 h after injury using EDTA and heparin (Fa. Sarstedt AG &Co. KG, Nümbrecht, Germany) as anticoagulants. Samples were centrifuged at room temperature at 1,882 g for 5 min. The resulting plasma was aliquoted and stored at -80 °C until further analysis.

### Cytokine array analysis and identification of up- and down- regulated proteins

Plasma samples from patients with TBI, SCI, and MT, as well as pooled plasma from healthy volunteers, were analyzed using Proteome Profiler Human XL Cytokine Array Kit (R&D Systems, Bio-Techne, Wiesbaden, Germany), according to the manufacturer’s instructions. This membrane-based sandwich immunoarray enables semiquantitative detection of 105 different cytokines. Capture antibodies and controls are spotted in duplicate on a nitrocellulose membrane. The plasma samples were not pooled but analyzed individually for each patient of the TBI, SCI, and MT groups. Only the norm group was pooled. In total 9 arrays were performed per group without a replicate.

Membranes were first blocked in array buffer for 1 h on a rocking platform shaker. Following blocking, 100 µl of plasma diluted in a final volume of 1.5 ml array buffer was incubated with the membrane overnight at 4 °C. The next day, membranes were incubated with detection antibody cocktail (30 µl diluted in 1.5 ml array buffer) for 1 h at room temperature under continuous rocking. Streptavidin-HRP and chemiluminescent detection reagents (1 ml Chemi Reagent Mix 1 and 2) were then applied.

Chemiluminescent signals were detected using a CCD-based imaging system (Amersham Imager 600 RGB, GE Healthcare Bio-Science, Uppsala, Sweden). Raw data were processed in a series of steps including background subtraction, spot intensity quantification, and normalization to internal reference spots, using ImageQuant TL 8.1 software (GE Healthcare Life Sciences).

To identify proteins relevant to TBI, SCI, or MT, corrected intensities were log2 transformed (a doubling translated to fold change + 1, a reduction of 50% to fold change − 1). A fixed log2 fold change cut-off (≥ 1.0 / ≤ -1.0 fold) was used to identify up- and down-regulated proteins. The fold change was defined as the ratio of spot intensities between norm group and each patient group (TBI, SCI, MT) under comparison. To identify overlap proteins in the three patient groups, Venn diagrams were generated.

### Osteogenic differentiation of human mesenchymal stem cells

The participation of each patient was communicated to our cell laboratory at least 24 h in advance to ensure the availability of mesenchymal stem cells (MSC). MSC (Fa. Lonza, Basel, Switzerland) were plated at a density of 1 × 104 cells / well in 24-well plates (Fa. BD Biosciences, Franklin Lakes, NJ, USA) and cultured overnight in complete culture medium (RPMI1640 (Fa. Invitrogen, Waltham, MA, USA)) with 10% fetal calf serum (FCS (Fa. Sigma-Aldrich, St. Louis, MO, USA)). To analyze the effect of different blood plasmas on osteogenic differentiation of MSC, plasma was added, at concentrations of 25 µl, 50 µl, 100 µl, 200 µl and 250 µl/ml, to RPMI1640. Additionally, MSC were cultured with / without osteogenic medium (Fa. Invitrogen, Waltham, MA, USA). For osteogenic differentiation assays low passage MSC (4–7 passages) were used. After 2 weeks of incubation, cells were analyzed for osteogenic differentiation using Alizarin red S staining. The medium was not changed during the differentiation period, and the plasma was added to the MSCs directly after preparation (at latest 2 h after blood collection).

### Statistical analysis

Statistical analysis between patient groups was performed using one-way analysis of variance (ANOVA) with correction for multiple comparisons (Bonferroni). A p-value ≤ 0.05 was considered statistically significant. This statistical comparison allowed the detection of differentially expressed proteins among the three patient groups. The data were generally expressed as median from at least nine independent patients or norms per group. Statistica 13.0 software was used for statistical evaluation of data. Due to the limited group sizes, a correlation between the cytokine array data and the in vitro findings was not performed.

## Results

### Demographic and clinical characteristics of patients with TBI, SCI and MT

The demographic and clinical characteristics of patients with TBI, SCI, and MT are shown in Tables 1, 2 and 3, respectively.

**Table 1:** Demographic and clinical characteristics of patients with TBI

Characteristic TBI (*n* = 9)	Data
**Age (mean ± SD)**	mean ± SD (median = 69) 59 ± 22
**Men / women (n)**	6 / 3
**Glasgow Coma Score (range)**	3–15
**Injury Severity Score (range)**	25–38
**Classification** Mild / moderate / severe (n)	- / 1 / 8
**Type of injury (n)**	
Contusion, subdural haematoma Contusion, subarachnoid haemorrhage Subdural, epidural haematoma Subdural, subarachnoid haemorrhage Subdural, subarachnoid contusion	5 1 1 1 1
**Side of injury (n**)	
Left Right Bilateral	4 1 4
**Area of injured brain (n)**	
Frontal, temporal parietal Frontal parietal Temporal parietal Occipital parietal Cerebellum Brain stem	4 2 1 2 - -
**Associated injury (n)**	
1 fracture 2 fractures 3 or more fractures	6 2 1
**Co-morbidity**	
1 disease 2 diseases 3 or more diseases	1 - 5
**Injury mechanism**	
Road traffic accidents Fall Sports Other	3 6 - -
**Hospitalization**	
Number of days (mean ± SD)	mean ± SD (median = 19) 45 ± 44
Development **of HO (n)**	
Yes No	1 8

**Table 2:** Demographic and clinical characteristics of patients with SCI

Characteristics SCI (*n* = 9)	Data
Age (mean ± SD)	mean ± SD (median = 55) 49 ± 7
Men / women (n)	7 / 2
Glasgow Coma Score (range)	3–15
Injury Severity Score (range)	25–57
**Classification SCI** Complete / incomplete / total	5 / 4 / -
**Injury mechanism (n)**	
Road traffic accidents Bicycle accidents High and low falls Sports Other	5 2 2 - -
**Spine level injury (n)**	
Cervical Thoracic Lumbar	5 4 -
**Trauma class (n)**	
Blunt	9
**Associated injury (n)**	
Spinal fracture Fracture of other parts 1 fracture 2 fractures 3 or more fractures	3 2 1 4
**Co-morbidity (n)**	
1 disease 2 diseases 3 or more diseases	1 2 -
**Hospitalization**	
Number of days (mean ± SD)	mean ± SD (median = 93) 76 ± 36
**Signs of HO (n)**	
Yes No	6 3

**Table 3:** Demographic and clinical characteristics of patients with MT

Characteristics MT (*n* = 9)	Data
**Age (mean ± SD)**	mean ± SD (median = 47) 40 ± 13
**male / female (n)**	5 / 4
**Glasgow Coma Score (range)**	3–15
**Injury Severity Score (range)**	18–50
**Trauma Class**	
Blunt Penetrating Other	9 - -
**Injury mechanism (n)**	
Road traffic accidents Train accidents Fall Sports Other	8 1 - - -
**Amount of fractures (n)**	
2 fractures 3 fractures 4 or more fractures	1 4 4
**Injured body regions (n)**	
Head Thorax Spine Extremity Pelvis Abdomen Other	1 6 3 7 6 6 -
**Co-morbidity (n)**	
1 disease 2 diseases 3 or more diseases	2 - -
**Hospitalization**	
Number of days (mean ± SD)	mean ± SD (median = 42) 34 ± 15
**Signs of HO (n)**	
Yes No	- 5

### Analysis of protein expression data

The evaluation of three different patient groups (TBI, SCI, MT) and 105 proteins resulted in a wide coverage of fold changes, ranging from no change to a fold change of 19.7. Therefore, fold changes were log2 transformed for further data-analysis.

For each patient group significant proteins were selected by log2 fold change ≥ 1 or ≤ -1 (*p* ≤ 0.05), respectively. A log2 fold change ≥ 1 was considered as an up-regulated protein, while log2 fold change ≤ -1 was considered as a down-regulated protein.

Cytokine array analysis showed that the expression levels of 98 proteins were comparable to the norm group. Based on cut-off criteria, seven proteins were identified as significant different between patients and healthy volunteers, including 4 up-regulated and 3 down-regulated proteins. The expression levels of these proteins are shown in Table 4. TBI induced up-regulation of four proteins and SCI of two proteins. A decrease in the expression levels of three proteins and an increase of one protein were observed after MT.

**Table 4:** Differentially expressed proteins between patients and normal controls. List of significantly up-regulated (↑) and down-regulated (↓) proteins (log2 fold change ≥ 1/ ≤ -1). Additionally, data are presented as median and interquartile range (IQR)

			Expression level						
			Median (IQR)				(log_2_ fold change ≥ 1/ ≤ -1) (*p* ≤ 0.05)		
Protein	Gene ID	Desciption / Protein class	Norm	TBI	SCI	MT	TBI	SCI	MT
GDF-15	9518	Growth Differentiation Factor 15 / signal peptide	**8.77** **(2.61)**	**29.52** **(33.87)**	**18.23** **(18.15)**	**22.28** **(16.67)**	**1.75 ↑**	**1.06 ↑**	
Growth hormone	2688	Somatotropin / growth factor, peptide hormone	**1.26** **(1.26)**	**6.73** **(6.90)**	**11.07** **(13.32)**	**9.11** **(22.47)**	**2.42 ↑**	**3.14 ↑**	
IL-2	3558	Interleukin 2 / cytokine	**2.41** **(1.18)**	**1.02** **(0.75)**	**0.72** **(1.36)**	**0.75** **(0.65)**			**-1.68 ↓**
IL-6	3569	Interleukin 6 / cytokine	**1.14** **(0.46)**	**0.74** **(0.49)**	**0.00** **(0.74)**	**0.45** **(0.48)**			**-1.34 ↓**
Leptin	3952	Leptin, peptide hormone	**2.14** **(0.84)**	**35.94** **(12.92)**	**1.10** **(1.78)**	**6.98** **(15.91)**	**4.07 ↑**		
RANTES	6352	C-C Motif Chemokine Ligand 5 (CCL5) / chemokine	**51.15** **(5.44)**	**42.24** **(40.24)**	**31.87** **(43.24)**	**21.64** **(19.03)**			**-1.24 ↓**
ST2, IL1RL1	9173	Interleukin-1 Receptor-like 1 / signal peptide	**5.64** **(1.78)**	**12.86** **(9.35)**	**10.58** **(12.67)**	**14.55** **(11.21)**	**1.19 ↑**		**1.37 ↑**

Only four proteins were statistically differentially up-regulated between groups. These elevated proteins included Growth Differentiation Factor 15 (GDF15), growth hormone, leptin and suppression of tumorgenicity 2 (ST2).

**Fig. 1:**
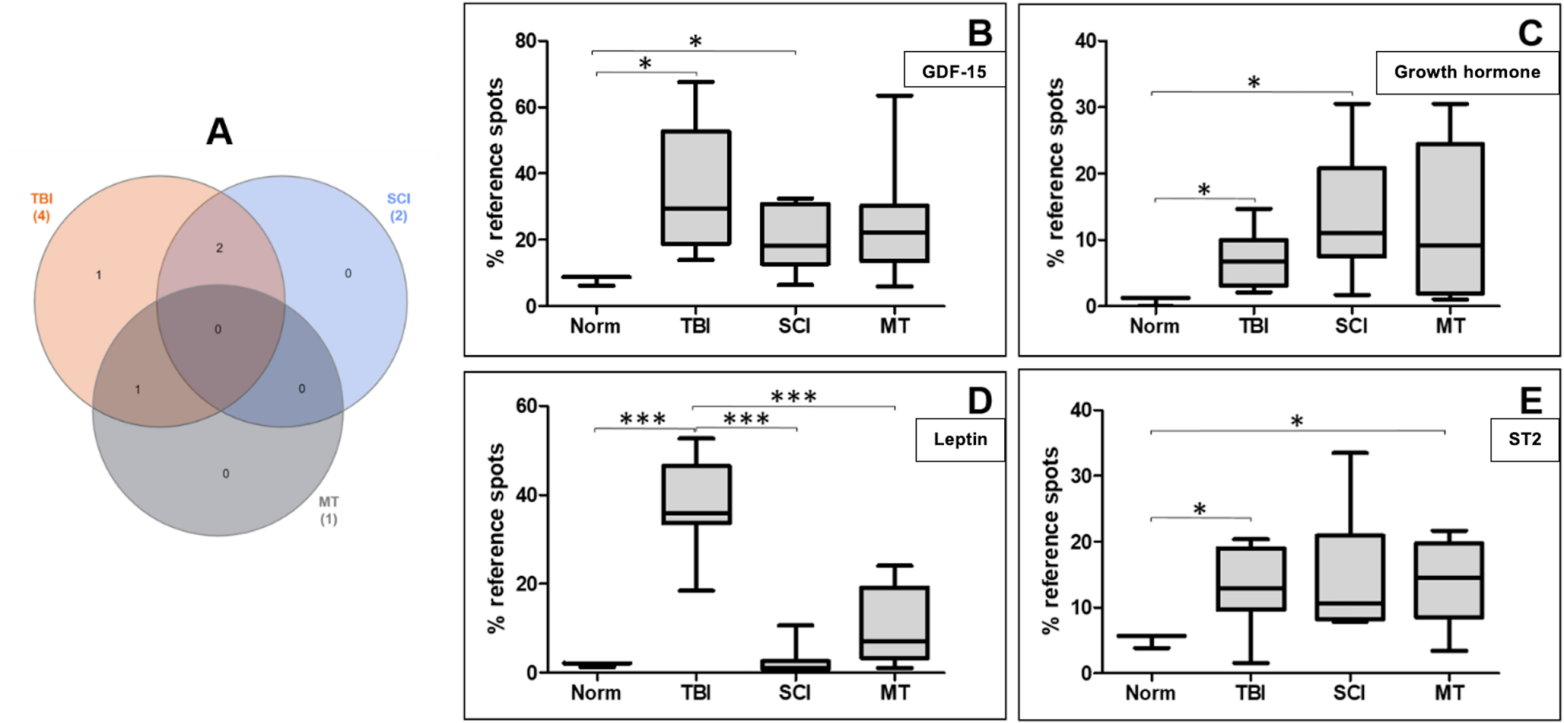
(**A**) Venn diagram shows the distribution of differentially expressed blood plasma proteins in response to TBI (red), SCI (blue) and MT (grey). Up-regulated proteins were selected by log2 fold change ≥ 1 and statistical significance (ANOVA *p* < 0.05). (**B**) Box whisker plots of proteins that showed a significant group difference (ANOVA / Bonferroni’s Multiple Comparison Test: *p* < 0.05 *; *p* < 0.001 ***). The four groups (norm, TBI, SCI and MT) are arranged along the x-axis and the normalized signal intensity of GDF-15 (**B**), growth hormone (**C**), leptin (**D**) and ST2 (**E**) are shown on the y-axis. The outer edges of the box represent the 25% quartile as well as the 75% quartile. The black line marks the respective median of the data. The whiskers indicate the minimum and maximum of the signal intensities

To determine whether the three patient-groups showed similarities in protein expression changes, a Venn diagram was generated from these significant up-regulated proteins (Fig. 1A). Venn diagram analysis represents the number of proteins that overlap between the patient groups (SCI, MT, TBI). Two up-regulated proteins overlapped between TBI- and SCI-group and one up-regulated protein between TBI and MT, respectively (Fig. 1A). Figure 1B shows, that the protein expression of plasma GDF-15 levels was significantly up-regulated in TBI- and SCI-group in contrast to norm-group (*p* < 0.05). The expression of growth hormone was also significantly higher than norm-group (TBI: *p* < 0.05 and SCI: *p* < 0.05) (Fig. 1C). Furthermore, expression of leptin was significantly increased in TBI-group compared to other groups (TBI vs. Norm: *p* < 0.0001, TBI vs. SCI: *p* < 0.0001 and TBI vs. MT: *p* < 0.0001) (Fig. 1D). Significantly higher ST2 levels were found in TBI-group as well as in MT-group (TBI vs. Norm: *p* < 0.05 and MT vs. Norm: *p* < 0.05) (Fig. 1E).

### Osteogenic differentiation assay

**Fig. 2:**
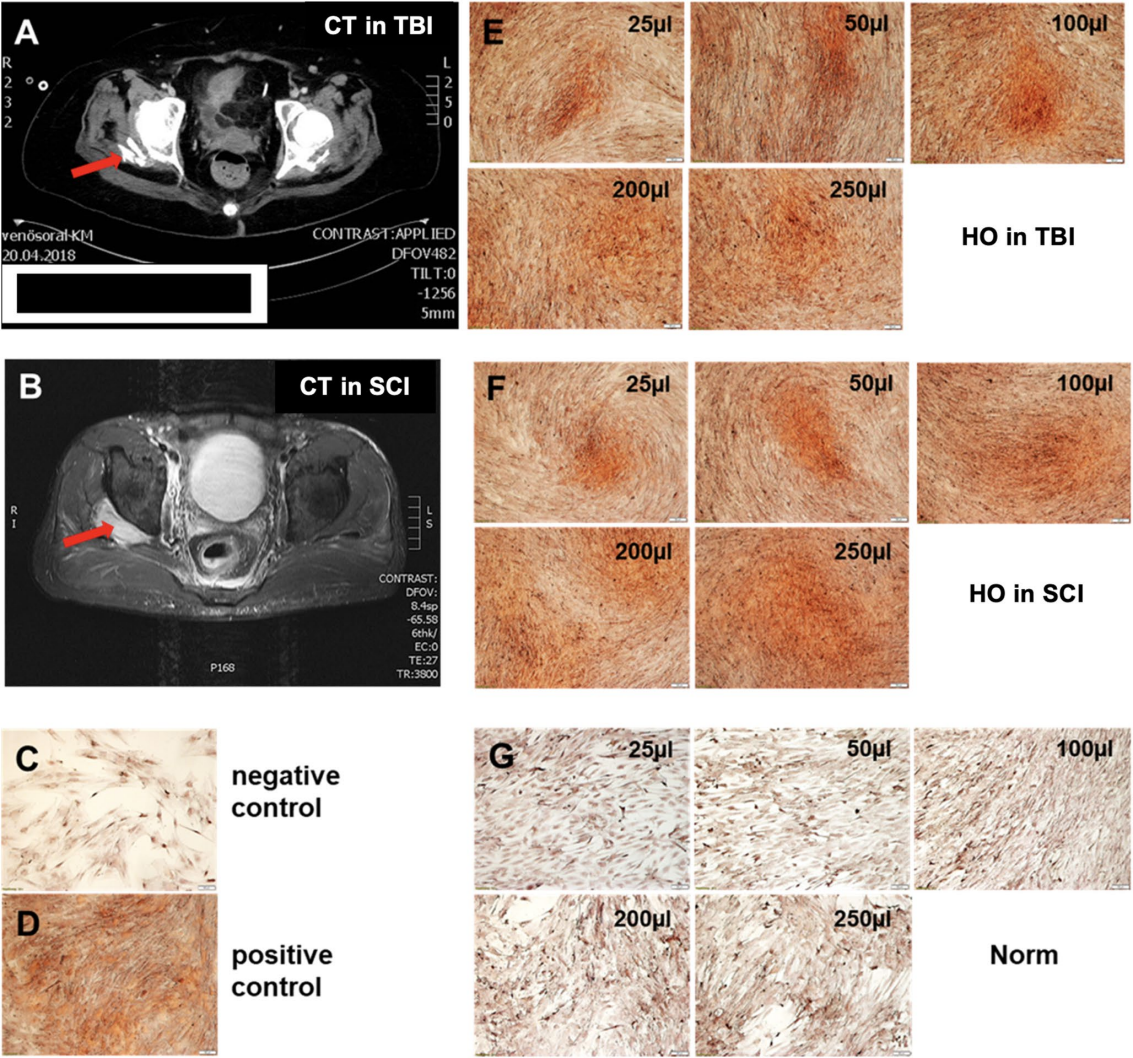
A 52-year-old female patient sustained TBI after road traffic accident. Computed tomography (CT) image was taken five months after injury and shows heterotopic bone on the right side (red arrow). **B**. A 27-year-old male patient suffered SCI after road traffic accident. Pelviabdominal magnetic resonance tomography (MRT) image was recorded nine days after injury and shows early signs of HO on the right side (red arrow). **E**-**G**. Effect of different patients´ blood plasma on osteogenic differentiation of MSC after an incubation time of 14 days. **E**-**F**. Incubation in the presence of different plasma volumes of patients with clinical signs of HO following TBI (**E**) or SCI (**F**). **G**. Incubation in norm-serum. **C**-**D**. Negative (cell culture medium) and positive (osteogenic medium) control of osteogenic differentiation. Mineralization of MSC was evaluated by Alizarin red S staining. Magnification: ×10; scale bar: 100 μm

In order to investigate the osteoinductive potential of plasma of patients with TBI or SCI, osteogenic differentiation assays using human MSC were performed. As is shown in Fig. 2E-F, MSC treated with blood plasma of patients with clinical signs of HO following TBI or SCI, exhibited a large amount of mineralized areas already after addition of 25 µl plasma, which increased continuously with increasing plasma concentrations. MSC were intensively stained with Alizarin red S staining, indicating the high degree of mineralization, as can be observed by comparing with the negative and positive osteogenesis controls (Fig. 2C-D). In contrast, no Alizarin red positive areas were found after treatment with norm-plasma (Fig. 2G).

**Fig. 3:**
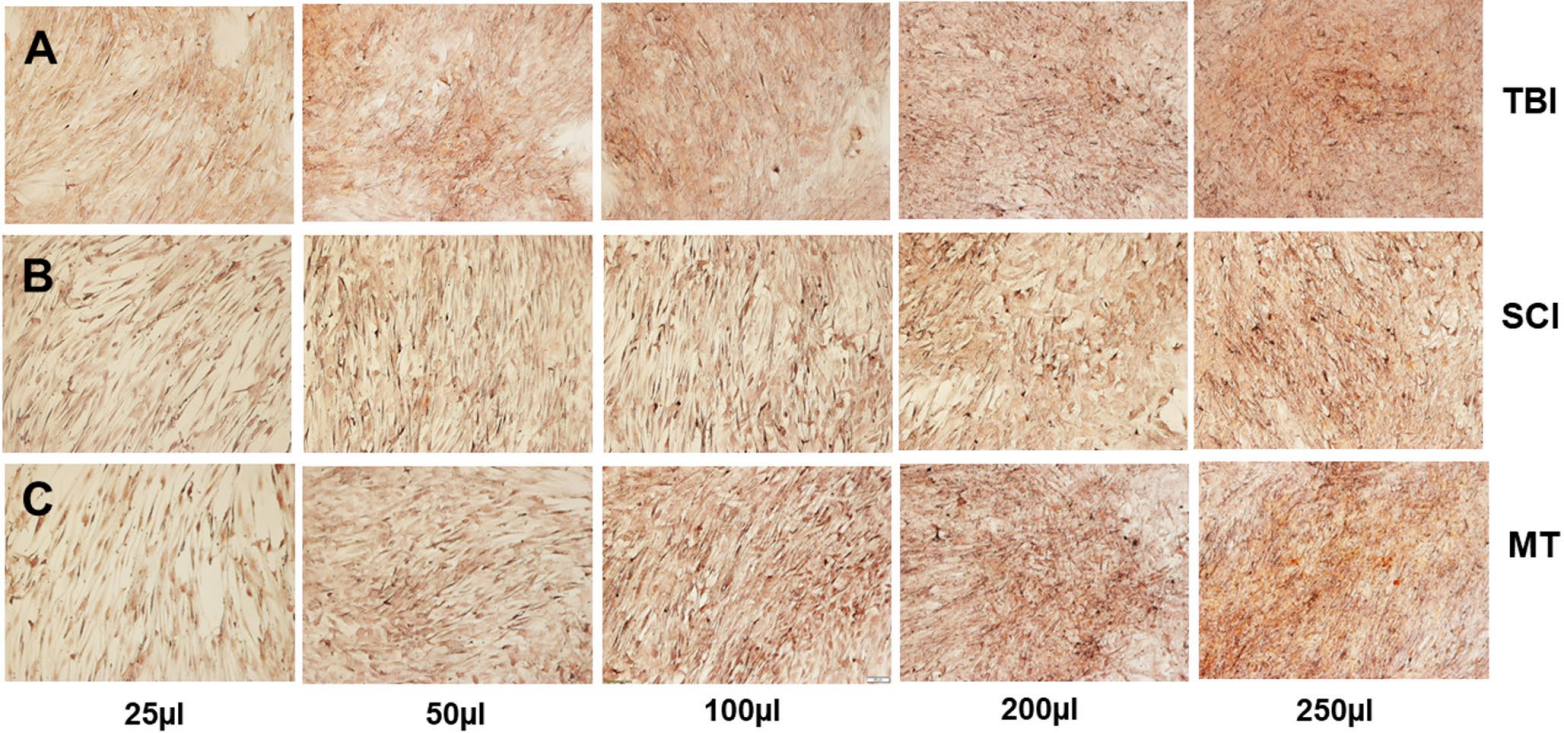
Effect of different patients´ blood plasma on osteogenic differentiation of MSC after an incubation time of 14 days. Incubation in the presence of different plasma volumes of patients without clinical signs of HO following TBI (**A**), SCI (**B**) or MT (**C**). Mineralization of MSC was evaluated by Alizarin red S staining. Magnification: ×10; scale bar: 100 μm

Figure 3 clearly demonstrate that the osteogenic differentiation potential differed extremely between TBI- and SCI- groups with/without clinical signs of HO. Compared to plasma of patients without signs of HO (Fig. 3A-B), MSC exhibited an obviously stronger capacity for osteogenic differentiation after treatment with plasma of TBI- as well as SCI-group with clinical signs of HO (Fig. 2E-F). Additionally, only poor Alizarin red positive areas were found in 250 µl plasma of MT-group (3 C).

## Discussion

Our findings demonstrate that blood plasma from patients with TBI or SCI significantly promotes osteogenic differentiation in MSCs in vitro. This effect was especially pronounced in patients with clinical signs of HO, indicating the presence of circulating pro-osteogenic factors in early post-injury phase. To date, molecular mechanisms underlying NHO remain incompletely understood, and reliable biomarkers for early risk stratification are lacking [12]. By integrating cytokine array analyses and cell culture experiments, we observed that plasma proteins including GDF-15, growth hormone, leptin and ST2 of patient-derived plasma showed an upregulation in vitro. While these findings may support a potential association between altered plasma composition and enhanced osteogenic responses, they do not allow definitive attribution of the observed effects to specific plasma proteins, and additional plasma components may have contributed. Notably, this is the first study to correlate cytokine array findings with direct osteogenic effects in a cell-based model using patient-derived plasma samples, thus bridging the gap between molecular alterations and their functional consequences. These observations may provide a biological explanation for the clinical association between severe neurotrauma and HO development and suggest that simple in vitro assays may offer a future tool for individualized risk prediction; however, further studies are required to validate this approach.

Our findings are in line with earlier studies demonstrating a link between neurogenic injury and increased risk of HO [13]. However, most previous work has focused on histological or imaging-based endpoints [14]. Functional in vitro approaches using patient-derived plasma have rarely been applied in this context. Several of the proteins found to be upregulated in our patient plasma samples, such as GDF-15, growth hormone, leptin and ST2, have previously been implicated in the pathogenesis of HO or in related osteogenic and inflammatory pathways. Notably, experimental work has shown that sensory nerves can express osteoinductive signals, suggesting a potential neural contribution to dysregulated bone formation after neurotrauma [15]. 

Uchiyama et al. investigated the effects of GDF-15 on human bone marrow-derived mesenchymal stem cells (BM-MSC) in the context of primary myelofibrosis. In their in vitro experiments, BM-MSC were cultured with recombinant GDF-15 for four weeks. They showed that GDF-15 promoted osteogenic differentiation, as evidenced by positive Alizarin red S staining indicating mineralized matrix deposition, which was absent in untreated control cells [16]. 

Previous studies have identified growth hormone as a factor that supports osseous tissue development by promoting osteogenic differentiation [17]. Moreover, elevated serum levels of growth hormone have been observed in patients following TBI showing enhanced osteogenesis, suggesting a potential link between neurotrauma and a systemically pro-osteogenic state [18]. 

Thomas et al. were able to show that leptin acts on human bone marrow stromal cells by promoting osteoblast differentiation while simultaneously inhibiting adipocyte lineage commitment [19]. These findings align with our observation of elevated leptin levels in patient plasma and support the hypothesis that leptin may contribute to a systemically pro-osteogenic environment in the early phase following trauma. Furthermore, leptin has been shown to influence the development of HO by promoting osteogenic differentiation of tendon derived stem cells and enhancing ectopic bone formation. This effect is mediated through activation of the mTORC1-signaling pathway, as demonstrated in both in vitro and in vivo models [20]. These findings are consistent with our results, in which elevated leptin levels were detected in patient plasma, further supporting its role in pathogenesis of HO. In a study by Wang et al., leptin was shown to influence bone metabolism by promoting osteogenic differentiation and enhancing callus formation. Using a rat model combining TBI and femoral fracture, the authors observed significantly elevated leptin levels both systemically and at the fracture site, accompanied by increased callus formation [21]. These finding align with our observation of elevated leptin concentrations in patients with neurotrauma and support the notion that leptin may contribute to a systemically osteo-inductive state involved in the development of HO.

While there are currently no published studies directly linking ST2 to the pathogenesis of HO, the IL-33/ST2 axis is known to play significant roles in inflammation, fibroblast activation, and immune cell recruitment following tissue injury, all of which are known to contribute to the development of HO [22–24]. 

The main limitation of this study is the limited sample size, which may restrict the statistical generalizability but does not compromise the observed trend. Due to the limited group sizes, a correlation between the cytokine array data and the in vitro findings was not performed; however, such correlations would be of interest for future studies. Therefore, the observed findings should be interpreted as notable patters rather than as causal relationships in the development of HO. In addition, while the control group was age-matched, matching for comorbidities was not feasible and thus represents a limitation of the study. Moreover, clinical factors such as medication use, sedation, or ventilation could potentially influence plasma protein expression. While these variables were not the primary focus of this investigation, they reflect the complexity of the clinical setting and highlight the need for further controlled studies with larger cohorts, specifically designed to investigate these factors.

## Conclusion

Our findings demonstrate a diverse pattern of plasma protein expressions in patients with TBI, SCI and MT without CNS involvement during the early post-injury phase. The occurrence of NHO was associated with enhanced osteogenic differentiation of MSC upon exposure to patient-derived plasma. These results suggest that a standardized MSC-based differentiation assay may represent a potential tool for early risk stratification of HO development using patient blood samples. However, these findings do not allow causal conclusions, and further studies are required to clarify the underlying mechanisms and the predictive value of the assay.

## Data Availability

No datasets were generated or analysed during the current study.

## Abbreviations

TBI: Traumatic brain injury
SCI: Spinal cord injury
MT: Multiple trauma
HO: Heterotopic ossification
NHO: Neurogenic heterotopic ossification
MSC: Mesenchymal stem cell
GDF15: Growth differentiation factor 15
ST2: Suppression of tumorigenicity 2
IL: Interleukin
ISS: Injury Severity Score
GCS: Glasgow Coma Scale
CT: Computed tomography
MRI: Magnetic resonance imaging
CCD: Charge–coupled device
EDTA: Ethylenediaminetetraacetic acid
